# Neonatal outcomes among twins born through assisted reproduction, compared to those born naturally

**DOI:** 10.1097/MD.0000000000040630

**Published:** 2024-11-22

**Authors:** Lin Lin, Ting Yao, Qiuping Liao, Jiaoxia Liu, Liping Huang, Lianghui Zheng

**Affiliations:** a Fujian Maternity and Child Health Hospital College of Clinical Medicine for Obstetrics & Gynecology and Pediatrics, Fujian Medical University, Fuzhou, Fujian, China; b Fujian Medical University, Fuzhou, Fujian, China.

**Keywords:** assisted reproductive technology (ART), intrauterine growth restriction, low birth weight, preeclampsia, premature birth

## Abstract

The growing prevalence of assisted reproductive technology (ART) is leading to a continuous rise in twin pregnancies. This study assessed the influence of ART on neonatal outcomes of twin pregnancies. Clinical records of twin deliveries at Fujian Maternity and Child Health Hospital between 2019 and 2021 were retrospectively selected and grouped based on the method of conception: ART-conceived and naturally conceived. Neonatal outcomes of interest included low birth weight (LBW), intrauterine growth restriction (IUGR), prematurity, low Appearance, Pulse, Grimace, Activity and Respiration scores, and neonatal intensive care unit admission. Univariate and multivariable logistic regression analyses were conducted to adjust for potential confounders. The results were expressed as odds ratio (OR) with 95% confidence interval (CI). A total of 1270 pairs of twins were included in the analysis. ART-conceived twins had significantly lower odds of being born with LBW (adjusted OR 0.57, 95% CI: 0.43, 0.75), prematurity (adjusted OR 0.71, 95% CI: 0.55, 0.91), and IUGR (adjusted OR 0.21, 95% CI: 0.10, 0.39) compared to naturally conceived twins. Rates of other outcomes were comparable in both groups. Twins born through ART had reduced odds of LBW, prematurity, and IUGR, with no impact on other neonatal outcomes. These findings suggest that ART may have a protective effect on twin fetal growth, although the risks associated with multiple gestations remain. Further studies should explore the mechanisms and long-term effects of these outcomes.

## 1. Introduction

The continuous development of assisted reproductive technologies (ART), such as in vitro fertilization (IVF), intracytoplasmic sperm injection (ICSI), embryo cryopreservation, etc, has revolutionized the field of reproductive medicine, providing new opportunities for couples facing infertility.^[1]^ The increasing use of ART led to a corresponding rise in the number of multiple pregnancies, particularly twin births, due to the common practice of transferring multiple embryos during IVF,^[2,3]^ raising concerns regarding its potential impact on neonatal outcomes in multiple pregnancies, which already carry higher risks of complications compared to singleton pregnancies.^[4–6]^

Twins born through ART are subjected to unique biological and environmental factors, such as the underlying infertility of the parents, the use of fertility medications, and the manipulation of embryos outside the natural maternal environment.^[7,8]^ These factors could potentially influence fetal development and neonatal outcomes. Therefore, understanding the differences in neonatal outcomes between twins born through ART and those conceived naturally is crucial for guiding clinical practice and counseling prospective parents.

Although some studies have suggested that ART-conceived twins may face higher risks of adverse outcomes, the evidence remains mixed, with variations based on factors such as the type of ART used, maternal age, and the presence of underlying maternal health conditions.^[9–11]^ This study aims to assess the potential impact of ART on neonatal outcomes of twins born through assisted reproduction.

## 2. Methods

### 2.1. Study population and eligibility

This retrospective cohort analysis compared neonatal outcomes of twins born through ART and conceived naturally. The study population included all twin deliveries recorded at Fujian Maternity and Child Health Hospital between 2019 and 2021.

Based on the method of conception, twins were classified into 2 groups. The ART-conceived group included neonates who were conceived using any form of ART, including IVF, ICSI, or the use of cryopreserved embryos. The naturally conceived group included all other twin pregnancies where conception occurred without medical intervention.

Inclusion criteria were as follows: (1) women with a confirmed twin pregnancy, (2) delivery at a gestational age of 24 weeks or greater, and (3) complete medical records available for both the mother and neonates.

Exclusion criteria were as follows: (1) monoamniotic twin pregnancies due to their unique risk profile, (2) cases where the method of conception was unclear, and (3) pregnancies with major congenital anomalies diagnosed prenatally or postnatally.

The study was approved by the Ethic Committee of Fujian Maternity and Child Health Hospital (No. 2024-KY197, Date: August 14th, 2024). The need for informed consent was waived in this retrospective study, and patient confidentiality was strictly protected by anonymization. The study adhered to the principles of the Declaration of Helsinki and relevant local regulations governing research with human subjects.

### 2.2. Data collection

Data were extracted from electronic medical records and included maternal demographics (age, parity, prepregnancy BMI, medical history), pregnancy characteristics (gestational age at delivery, mode of delivery), and neonatal outcomes. Key neonatal outcomes were low birth weight (LBW, defined as birth weight < 2500 g), intrauterine growth restriction (IUGR, defined as birth weight below the 10th percentile for gestational age), prematurity (defined as delivery before 37 weeks of gestation), low Appearance, Pulse, Grimace, Activity and Respiration (APGAR) score at 5 minutes (score <7), early neonatal mortality and admission to the neonatal intensive care unit (NICU).^[12]^ Data on maternal pregnancy complications (e.g., antenatal anemia, preeclampsia, gestational diabetes [GDM]) were also collected.

### 2.3. Statistical analysis

R version 4.4.1 was used. Categorical variables were expressed as frequencies and percentages. Continuous variables were reported as mean + standard deviation. A comparison of baseline variables was done using a chi-squared test for categorical variables and a *t* test for continuous variables. If 1 or both babies had the outcome of interest, it was coded as “Yes.” If the outcome was absent in both babies, it was coded as ‘No’ for the analysis. Univariate logistic regression was initially done to assess the association between the method of conception (ART vs natural conception) and each neonatal outcome. Unadjusted odds ratios (ORs) with 95% confidence intervals (CIs) were calculated for each outcome of interest. Multivariable logistic regression models were constructed for each neonatal outcome to account for potential confounders. Covariates included in the models were chosen based on clinical relevance and the principle of purposive selection.^[13,14]^ Each potential covariate was added to the univariate model individually, and the corresponding change in OR was noted. Covariates that caused at least a 15% change in the OR were included in the multivariable model, and the results were expressed as adjusted odds ratios (AORs) with 95% CIs. A two-sided *P* < .05 was statistically significant for all analyses.

## 3. Results

This retrospective study included medical records of 1270 women with twin pregnancies. Of them, 625 had conceived through ART (i.e., in vitro fertilization, IVF or intrauterine insemination), and 645 had normal spontaneous conception. The baseline characteristics of both groups are summarized in Table 1.

**Table 1:** Baseline characteristics based on the mode of conception.

Characteristic	Natural, N = 645*	IVF/IUI, N = 625*	*P*-value†
*Educational status*			
Junior high school/secondary school and below	169 (26%)	203 (32%)	.081
High school/tertiary school	210 (33%)	194 (31%)	
Bachelor’s degree	237 (37%)	199 (32%)	
Master’s degree and above	29 (4.5%)	29 (4.6%)	
*Antenatal anemia*	171 (27%)	149 (24%)	.3
*Advanced age at conception (≥35 years*)	93 (14%)	133 (21%)	.001
*Chronic hypertension*	5 (0.8%)	8 (1.3%)	.4
*Prepregnancy BMI*			
Underweight (<18.5 kg/m²)	96 (15%)	75 (12%)	.3
Normal (18.5–24.9 kg/m²)	479 (74%)	482 (77%)	
Overweight or obese (≥25.0 kg/m²)	70 (11%)	68 (11%)	
*Cervical insufficiency*	23 (3.6%)	45 (7.2%)	.004
*Gestational age in weeks* *Mean (SD*)	35.89 (2.11)	36.08 (1.97)	.10
*Sex of the newborns*			
Both male	266 (41%)	182 (29%)	<.001
Both female	265 (41%)	151 (24%)	
Male and female	114 (18%)	292 (47%)	
*Number of previous births*			
Zero	322 (50%)	520 (83%)	<.001
One	253 (39%)	96 (15%)	
Two or more	70 (10.8%)	9 (1.4%)	
*Gestational diabetes mellitus*	145 (22%)	177 (28%)	.017
*Preeclampsia*	58 (9.0%)	77 (12%)	.054
*No history of previous preterm birth*	642 (100%)	619 (99%)	.3

IUI = intrauterine insemination, IVF = in vitro fertilization.

* Data presented as n (%), unless otherwise stated.

† Comparison of baseline variables between the 2 groups was done using chi-squared test for categorical and using *t* test for continuous variables.

As shown in Table 1, advanced maternal age, cervical insufficiency, sex of the newborns, parity, and GDM status significantly differed between the 2 groups (*P* < .05).

Mean (standard deviation) gestational age (in weeks) of ART-conceived and naturally conceived neonates was comparable (Table 1). On univariate regression analysis, the risk of LBW neonates (OR 0.75, 95% CI: 0.59, 0.95) and IUGR (OR 0.25, 95% CI: 0.13, 0.44) was statistically lower in the ART-conceived group compared the naturally conceived group (Table 2). The results of the multivariable regression analysis, adjusted for maternal education, antenatal anemia, advanced maternal age, prepregnancy BMI, cervical insufficiency, parity, GDM, and preeclampsia showed that the risk of having LBW (OR 0.57, 95% CI: 0.43, 0.75), born premature (OR 0.71, 95% CI: 0.55, 0.91) and having IUGR (OR 0.21, 95% CI: 0.10, 0.39) was significantly lower in the ART-conceived group (Table 3). There were no statistically significant associations between the mode of conception and other outcomes.

**Table 2:** Distribution of outcomes based on mode of conception and findings of univariate regression.

Outcomes		N (%)	OR	95% CI	*P*-value
Low birth weight	Assisted	415 (33.2%)	0.75	0.59, 0.95	.017
	Normal	468 (36.3%)	Reference		
Intrauterine growth restriction	Assisted	13 (1.1%)	0.25	0.13, 0.44	<.001
	Normal	51 (4.0%)	Reference		
Premature birth	Assisted	323 (25.8%)	0.84	0.67, 1.04	.11
	Normal	362 (28.1%)	Reference		
Neonatal mortality	Assisted	4 (0.32%)	4.15	0.61, 81.3	.20
	Normal	1 (0.1%)	Reference		
Low APGAR score at 5 minutes	Assisted	2 (0.16%)	–	–	–
	Normal	0 (0.0%)	Reference		
Admission to NICU	Assisted	376 (30.1%)	1.0	0.80, 1.25	.99
	Normal	388 (30.0%)	Reference		

Odds ratio is estimated for IVF/IUI compared to natural conception (reference).

Number of neonates in assisted reproduction and normal conception were 1250 and 1290, respectively.

APGAR = Appearance, Pulse, Grimace, Activity and Respiration, CI = confidence interval, IUI = intrauterine insemination, IVF = in vitro fertilization, OR = odds ratio.

**Table 3:** Findings of multivariable regression for the association between mode of conception and neonatal outcomes in twin pregnancies.

Outcomes		OR	95% CI	*P*-value
Low birth weight	Assisted	0.57	0.43, 0.75	<.001
	Normal	Reference		
Intrauterine growth restriction	Assisted	0.21	0.10, 0.39	<.001
	Normal	Reference		
Premature birth	Assisted	0.71	0.55, 0.91	.008
	Normal	Reference		
Neonatal mortality	Assisted	–	–	–
	Normal	Reference		
Low APGAR score at 5 minutes	Assisted	–	–	–
	Normal	Reference		
Admission to NICU	Assisted	0.88	0.68, 1.13	.31
	Normal	Reference		

Odds ratio is estimated for IVF/IUI compared to natural conception (reference).

Adjusted for maternal education, antenatal anemia, advanced maternal age, pre pregnancy BMI, cervical insufficiency, parity (number of previous births), gestational diabetes mellitus, preeclampsia.

APGAR = Appearance, Pulse, Grimace, Activity and Respiration, CI = confidence interval, IVF = in vitro fertilization, OR = odds ratio.

## 4. Discussion

This study provides important insights into the neonatal outcomes of twin pregnancies achieved through ART. Twins conceived through assisted reproduction had reduced odds of LBW, being premature, and IUGR compared to naturally conceived neonates. Other key outcomes, such as APGAR scores, neonatal mortality, and admission to the NICU, were similar in both groups.

The observation that ART-conceived twins had lower odds of LBW, prematurity, and IUGR compared to naturally conceived twins is an intriguing finding that contrasts with some existing literature, which often associates ART with higher risks of adverse neonatal outcomes.^[4,5,9,10]^ One possible explanation for our findings might be the rigorous prenatal care and monitoring typically provided to women undergoing ART.^[15–17]^ These patients are often subject to more frequent and detailed medical evaluations, allowing for early detection and management of potential complications. Additionally, ART procedures often involve the selection of high-quality embryos,^[18,19]^ which may contribute to better fetal growth outcomes. Another important factor to consider is our exclusion of pregnancies with major congenital anomalies in this analysis. Some ART-conceived twins may have had such anomalies and were excluded from the study early on, which might have contributed to the lower odds of LBW, prematurity, and IUGR observed in this group. Furthermore, it is possible that the underlying causes of infertility in couples opting for ART do not necessarily impair fetal growth, particularly in cases where infertility is related to factors such as male infertility or tubal blockage rather than maternal conditions like obesity or chronic disease. This could result in a lower risk of LBW, prematurity and IUGR among ART-conceived twins compared to those conceived naturally, where such maternal factors may be more prevalent. Further studies that would stratify the cohort of ART pregnancies into groups based on the type of infertility may provide more insight.

The absence of significant differences between ART-conceived and naturally conceived twins in terms of low APGAR scores, mortality, and NICU admission is consistent with the idea that while ART may influence some neonatal outcomes, it does not universally increase the risk of all outcomes.^[5,20]^ Our findings suggest that the risks traditionally associated with twin pregnancies are primarily driven by the nature of multiple gestations rather than the method of conception. The comparable rates of low APGAR scores and NICU admissions between ART-conceived and naturally conceived twins also suggest that the immediate postnatal adaptation and need for intensive care are not significantly influenced by the mode of conception.

This study has some strengths that need to be acknowledged. Our sample size was large, and a comprehensive range of neonatal outcomes was studied. Additionally, using multivariable regression analysis to adjust for potential confounders strengthens the validity of our conclusions, minimizes bias, and offers a clearer picture of the link between ART and neonatal outcomes.

However, the study also has limitations. Firstly, the study is limited in its ability to establish causality, that is, the associations identified may not necessarily imply that ART is the direct cause of the observed outcomes. Secondly, there is the potential for selection bias, as women undergoing ART often receive more intensive prenatal care, which could contribute to better outcomes and skew the comparison with naturally conceived twins. Thirdly, the heterogeneity of ART procedures, which include various techniques such as IVF and ICSI, was not accounted for in the analysis, limiting the ability to conclude the effects of specific ART methods. Furthermore, while several important confounders were controlled for, data on other potential influencing factors, such as maternal nutrition and pregnancy weight gain, were not included. Finally, the generalizability of the findings may be limited, as cultural, socioeconomic, and healthcare system differences in other regions could influence the outcomes of ART-conceived twins.

### 4.1. Clinical implications and future research

Our findings carry significant clinical implications for the management of twin pregnancies conceived through ART. The reduced risk of LBW, prematurity, and IUGR in ART-conceived twins may provide some reassurance to prospective parents and healthcare providers regarding the potential benefits of ART in achieving favorable outcomes. However, the persistent risks of other neonatal complications underscore the need for continued vigilance and comprehensive prenatal care in all twin pregnancies, regardless of the method of conception. Studies that explore the impact of specific ART techniques, such as single vs multiple embryo transfer or the use of cryopreserved embryos, on neonatal outcomes could provide valuable insights into optimizing ART protocols for twin pregnancies. Additionally, research into the long-term developmental outcomes of ART-conceived twins compared to those conceived naturally would provide a more comprehensive understanding of the potential implications of ART on child health and development.

## 5. Conclusion

In conclusion, our study highlights a reduced risk of LBW, prematurity, and IUGR among ART-conceived twins, with no significant differences in other key neonatal outcomes. These findings suggest that ART might have beneficial effects on fetal growth in twin pregnancies, though the inherent risks of multiple gestations remain. Our results underscore the importance of tailored prenatal care for twin pregnancies and provide a foundation for future research to optimize outcomes for all twins, regardless of their mode of conception.

## Author contributions

**Conceptualization:** Lin Lin.

**Data curation:** Ting Yao, Qiuping Liao, Jiaoxia Liu, Liping Huang, Lianghui Zheng.

**Formal analysis:** Ting Yao, Qiuping Liao, Jiaoxia Liu.

**Funding acquisition:** Lin Lin, Qiuping Liao, Lianghui Zheng.

**Investigation:** Qiuping Liao, Jiaoxia Liu.

**Methodology:** Ting Yao, Qiuping Liao, Jiaoxia Liu, Liping Huang, Lianghui Zheng.

**Project administration:** Lin Lin, Liping Huang, Lianghui Zheng.

**Supervision:** Lin Lin, Liping Huang.

**Validation:** Ting Yao, Qiuping Liao.

**Visualization:** Liping Huang, Lianghui Zheng.

**Writing – original draft:** Lin Lin, Liping Huang, Lianghui Zheng.

**Writing – review & editing:** Lin Lin.
